# ARGscape: A modular, interactive tool for manipulation of spatiotemporal ancestral recombination graphs

**Published:** 2025-10-08

**Authors:** Christopher A. Talbot, Gideon S. Bradburd

**Affiliations:** 1 Department of Ecology and Evolutionary Biology, University of Michigan, 1105 North University Avenue, Ann Arbor, MI 48109, USA; 2 Department of Computational Biology, Cornell University, 350 Tower Rd, Ithaca, NY 14853, USA

## Abstract

**Summary:**

Ancestral recombination graphs (ARGs) encode the complete genealogical history of a population of recombining lineages. ARGs, and their succinct representation, tree sequences, are increasingly central to modern population genetics methods, yet building an intuition for ARGs remains challenging. This is particularly true when analyzing ancestry in a geographic context, as there is a critical lack of dedicated, interactive tools capable of visualizing ARGs as spatiotemporal objects. To address this gap, we introduce ARGscape, an interactive platform for simulating, analyzing, and visualizing ARGs across space and time. ARGscape provides a user-friendly graphical interface featuring dynamic 2- and 3-dimensional visualizations to explore ARGs through space and time, as well as a novel “spatial diff” visualization for quantitative comparison of geographic inference methods. ARGscape is an innovative, unified framework that seamlessly integrates leading command-line, Python, and R-based tools for ARG simulation, manipulation, and use in spatiotemporal inference into both graphical and command-line interfaces. By integrating these various functionalities, ARGscape facilitates novel data exploration and hypothesis generation, while lowering the barrier to entry for spatiotemporal ARG analysis in both research and education use-cases.

**Availability and Implementation:**

ARGscape is built with a Python FastAPI backend and a React/TypeScript frontend. It is freely available as a live demo at https://www.argscape.com [v0.3.0] and as a Python package on PyPI (pip install argscape) [v0.3.0]. The source code and documentation are available on GitHub at https://github.com/chris-a-talbot/argscape.

## 1 Introduction

The study of genetic variation across time and space is fundamental to population genetics, providing insights into processes from disease outbreaks to species evolution [Beebe et al., 2013, Breistein et al., 2022, Rehmann et al., 2025]. The record of these processes can be read from the Ancestral Recombination Graph (ARG), which traces the complete genetic ancestry, including recombination and coalescent events, for a set of samples [Rasmussen et al., 2014, Lewanski et al., 2024, Brandt et al., 2024, Nielsen et al., 2025]. These structures can thereby provide unprecedented clarity in studies of genetic variation across continuous space and time, moving beyond discrete populations and timepoints and toward a representation of real-world evolutionary processes. Recent developments in ARG inference methods have made high-quality ARGs accessible for a wide range of datasets of varying size and quality [Kelleher et al., 2019, Speidel et al., 2019, Hubisz et al., 2020, Wohns et al., 2022, Zhang et al., 2023, Deng et al., 2024]. In particular, the succinct tree sequence data structure, implemented by tskit, has made the storage and analysis of very large ARGs computationally tractable [Kelleher et al., 2016, Ralph et al., 2020, Wong et al., 2024].

Consequently, ARGs are now central to a growing number of sophisticated inference methods [Hejase et al., 2022, Wohns et al., 2022, Tagami et al., 2024, Grundler et al., 2025]. In particular, a growing area of research is the use of ARGs to infer the spatial history of ancestors of a population [Wohns et al., 2022, Osmond & Coop, 2024, Grundler et al., 2025, Deraje et al., 2025]. However, despite their theoretical power, a broadly accessible and unified framework for interpreting ARGs as spatiotemporal objects remains elusive. This gap hinders their adoption by the broader genomics community. While existing tools (such as tsbrowse and tskit_arg_visualizer, [Karthikeyan et al., 2025, Kitchens & Wong, 2025]) provide powerful interfaces for inspecting the raw topology of and data stored in tree sequences and ARGs, they are not designed for the integrated spatial inference and visualization that is critical for many ecological and evolutionary questions.

To bridge this gap and enhance the accessibility of ARGs, we present ARGscape: an interactive platform for visualizing, exploring, and analyzing ARGs as spatiotemporal records of evolutionary history. ARGscape integrates simulation, analysis, and visualization into a single, intuitive workflow, making complex spatiotemporal analyses accessible to both researchers and students.

## 2 System and Features

ARGscape is a full-stack web application with a Python-based FastAPI backend and a React/TypeScript-based frontend, available as a publicly hosted website or a Python package for local deployment. Its workflow is designed to be modular and intuitive, guiding the user from data input to visualization and export. The backend design is intended to facilitate rapid integration of new algorithms and features, ensuring that developments in the field can be incorporated and made accessible quickly. The ARGscape Python package also features a command-line interface incorporating core functionality without the need to load the full web application.

### 2.1 Data Input and Simulation

Users can begin by either uploading existing tree sequences in . trees or compressed . tsz format or by simulating new ones. Custom spatial coordinates for nodes in the tree sequence can be supplied via .csv files. The simulation module uses msprime to generate tree sequences under a specified coalescent model [Kelleher et al., 2016, Nelson et al., 2020, Baumdicker et al., 2022]. For demonstrative purposes, ARGscape uses a multidimensional scaling algorithm on the genealogical distance matrix to produce “toy” spatial coordinates for simulated samples, which can be generated on a 2-dimensional grid or projected onto real-world maps.

For command-line interface users, tree sequences can be loaded and stored in temporary memory using the argscape_load command.

### 2.2 Spatiotemporal Inference

A core function of ARGscape is to provide a cohesive analytical environment that integrates multiple complex spatiotemporal inference tools, enabling direct comparison and hypothesis testing within a single interface. Once a tree sequence is loaded, users can perform:

- **Temporal Inference:** ARGscape supports the full tsdate workflow, including ARG pre-processing and temporal inference [Speidel et al., 2021].
- **Spatial Inference:** Given sample locations in continuous 1- or 2-dimensional space, ARGscape enables rapid inference of ancestral node locations in geographic space. Geographic inference may be performed using any of the following models:
  - **Wohns midpoint** [Wohns et al., 2022]
  - **Gaia** [quadratic or linear algorithms] [Grundler et al., 2025]
  - **FastGaia** (see Supplementary Information, Section S2)
  - **Sparg** [Deraje et al., 2025]

These analyses are initiated with simple button clicks in the user interface, abstracting away the need for complex command-line syntax and separate data formatting for each approach. ARGscape also offers static and interactive command-line methods for manipulating tree sequences, including persistent storage of tree sequences and the full suite of spatiotemporal inference methods, using the argscape_infer command.

### 2.3 Interactive Visualization

ARGscape offers multiple visualization styles, each featuring extensive interactivity, customization, and analysis capabilities. Before visualizing with any method, the user may select a subset of the sample nodes, genomic regions, or temporal regions to visualize. This is particularly useful for ARGs that extend deep in time, for which visualizing only recent ancestry may be most informative. Across all visualizations, recombination nodes are combined into single nodes with multiple node IDs, which enhances intuitive visualization and increases rendering speed. ARGscape offers three primary visualization modes for exploring tree sequences:

1. **2D Non-spatial View:** A force-directed graph rendered using D3.js displays the topological structure of the ARG. The force-directed graph visualization is fully customizable, featuring drag-and-drop node placement, toggleable node and edge labels, and a customizable color theme. To maximize interpretability, users can arrange sample nodes (tips) using multiple ordering algorithms specifically designed for tree sequences, including a novel “consensus” method that aggregates node orders across multiple local trees. Internal nodes and edges are then placed using a force simulation to ensure ad- equate spacing for visual clarity. Additionally, the dagre−d3 mode uses the dagre−d3 React library to place all nodes and edges using a path-crossing minimization algorithm. Users can interactively explore the graph by clicking nodes to view sub-ARGs or trace ancestral lineages.
2. **3D Spatial View:** A dynamic 3D plot rendered with deck.gl visualizes the ARG in geographic space, with time on the z-axis. The 3D spatial visualization is highly customizable, featuring toggleable node and edge labels, as well as a customizable color theme. The tool automatically detects the coordinate reference system (CRS) to display nodes on a unit grid or a world map. Users can upload custom shapefiles for bespoke geographic contexts. Interactive sliders for genomic and temporal windows enable powerful data exploration, allowing users to isolate and visualize how specific segments of ancestry have moved across space over time. Sample nodes that occur in identical locations and are +/ − 1 node ID away from each other are assumed to represent haplotypes from the same individual, and are therefore combined into a single node for clarity. This specialized visualization mode opens doors for novel hypothesis generation and data exploration, including the visual assessment of coalescent rate through both time and space.
3. **3D Spatial “Diff” View:** A dynamic 3D plot rendered with deck.gl visualizes the difference in spatial locations on nodes in two ARGs with otherwise identical structure. Nodes are placed at the midpoint of their locations in each of two different ARGs, with a bar indicating the difference in locations between ARGs. This tool is one-of-a-kind and highly useful for comparing inferred ancestral locations with simulated ground truth, or comparing inferred locations between multiple different inference methods.

### 2.4 Accessibility and Data Export

To cater to different user needs, ARGscape is available as both a public web application for easy access and testing, as well as a Python package for integration into local pipelines and resource-intensive use cases. Users can download any modified tree sequence (e.g., after spatial/temporal inference) in standard . trees or . tsz formats for further downstream analysis. Publication-quality images of any visualization can also be exported, facilitating integration into manuscripts, presentations, and educational materials.

## 3 Conclusion

ARGscape provides a much-needed, user-friendly platform that integrates the simulation, analysis, and spatiotemporal visualization of ancestral recombination graphs. By integrating powerful but complex inference tools in an intuitive interface and providing novel interactive visualizations, it significantly lowers the barrier to entry for researchers and students interested in exploring the full richness of ARGs. ARGscape simplifies the process of asking and answering questions about how ancestry unfolds across the genome over time and across geographic space. Future directions will focus on enhancing performance for BioBank-scale datasets and expanding the tool’s analytical capabilities to visualize key demographic processes through time, including migration and admixture. We also aim to further develop its utility as an interactive learning platform for population genetics education by incorporating guided tutorials and visual-based lessons.

## Supplementary Material

Supplement 1

## Figures and Tables

**Figure 1:**
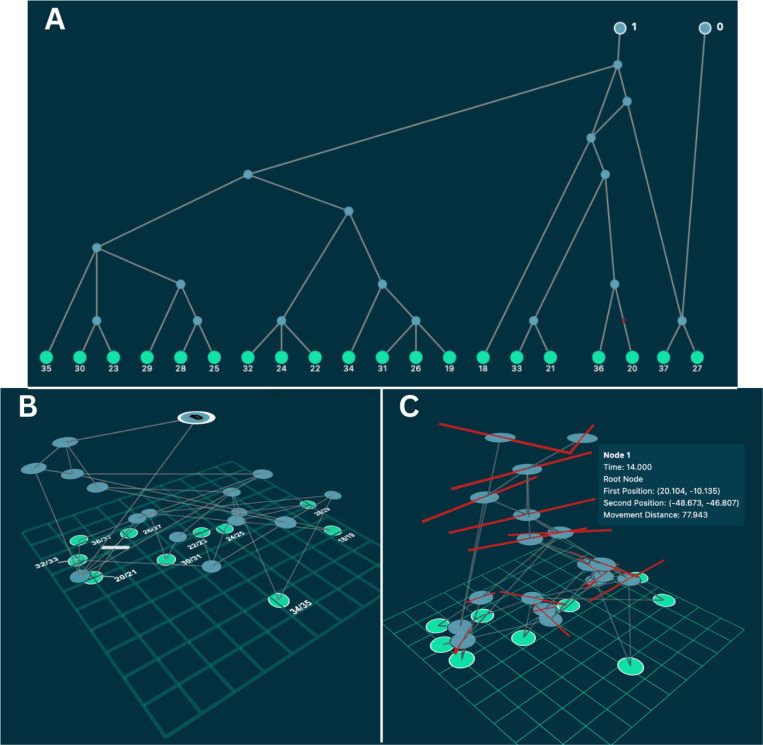
Visualizations of an ancestral recombination graph (ARG) generated by a spatially explicit evolutionary simulation in SLiM 5.0. **A)** A 2D visualization, displaying the topology of the ARG. Our dagre-d3 mode generates this node ordering and layout. Green nodes represent samples, and blue nodes represent ancestral nodes. Blue nodes with a white outline are roots of the ARG. Branches with mutations along them are marked with a red “x.” Generations are evenly spaced along the y-axis. In ARGscape, node locations may be adjusted, node and edge labels toggled, and colors modified. Nodes may be selected to display parent or child subARGs. **B)** A 3D visualization of the same ARG with ground truth spatial data from the simulation. Green nodes represent samples, and blue nodes represent ancestral nodes. Blue nodes with a white outline are roots of the ARG. Branches with mutations along them are marked with white bars. Generations are evenly spaced along the z-axis. Sample nodes from the same individual are labeled together because they originate from the same location. In ARGscape, spacing may be adjusted, node and edge labels toggled, and colors modified. Nodes may be selected to display parent or child subARGs. **C)** A 3D “spatial diff” visualization. Ancestral locations along the same ARG were re-inferred using the Gaia quadratic algorithm in ARGscape. Nodes are placed at the midpoint of the simulated and inferred locations, with red bars connecting the ground truth and inferred locations. This can be used to visualize the quantity and directionality of error in an inference method against ground truth; the increase in error moving back through time is clearly visible. Hovering over nodes reveals metadata, including their geographic locations in each version of the ARG and the distance between them.

## Data Availability

Source code and documentation are available at https://github.com/chris-a-talbot/argscape. Figure 1 uses a tree sequence generated by a spatially explicit simulation ran in SLiM 5.0 [Haller et al., 2025]. The tree sequence and SLiM simulation script used to generate visualizations for Figure 1 are available on Zenodo under doi 10.5281/zenodo.17296543.
