# Supplementary material for: ARGscape: A modular, interactive tool for manipulation of spatiotemporal ancestral recombination graphs
Source: ArXiv. 2025 Oct 8:arXiv:2510.07255v1. Preprint. [Version 1] (PMC12632698)
Supplement: Supplement 1 [file NIHPP2510.07255v1-supplement-1.pdf]

Supplementary Information

ARGscape: A modular, interactive tool for manipulation of spatiotemporal ancestral recombination graphs

Christopher A. Talbot[1,2,*] and Gideon S. Bradburd[1]

[1]Department of Ecology and Evolutionary Biology, University of Michigan, Ann Arbor, MI, USA

[2]Department of Computational Biology, Cornell University, Ithaca, NY, USA

[*]Corresponding author: cat267@cornell.edu

S1 Installation & Usage

S1.1 Using the Hosted Web Application

The latest production version of ARGscape is available at https://www.argscape.com/. The interactive interface guides users through the various modes of use and functionality, including file management, simulation, visualization, inference, and output downloading. Files stored on the hosted web server will be stored privately and securely for up to 24 hours. The web server storage is subject to being cleared, without notice, during updates.

We recommend using the hosted application only for **educational** and/or **exploratory** purposes due to the potential for data loss and the limited computational capabilities of our web server. Users are limited to a small amount of computing power for simulation, inference, and visualization, which will be insufficient for most full-scale projects.

S1.2 Installing ARGscape Locally

The latest production version of ARGscape is available as a Python package hosted on the Python Package Index (PyPI). To install, users must have Python version 3.8 or later. Then, from a terminal in which Python is available, users should run the following commands:

```
> python -m pip install --upgrade pip
> python -m pip install argscape
```

ARGscape will be installed in the Python environment.

S1.3 Running the Web Application Locally

To run the web application hosted on a local machine, first see Supplemental Information 1.2 – Installing ARGscape Locally. Once ARGscape is installed, the web application can be started from any terminal in which Python is available and ARGscape is installed using the following command:

```
> argscape
```

This command will launch the complete ARGscape web application in a browser window, hosted on your local machine. For additional options, including customizing the port ARGscape is hosted on and disabling certain features, run:

```
> argscape --help
```

S1.4 Using the Command-Line Tools

To run the web application hosted on a local machine, first see Supplemental Information 1.2 – Installing ARGscape Locally. Once ARGscape is installed, the command-line tools can be

used from any terminal in which Python is available and `ARGscape` is installed. The available commands include:

S1.4.1 argscape

The basic `argscape` command loads the web application in a browser window.

S1.4.2 argscape_load

The `argscape_load` command includes features for session file management, including loading tree sequences from files using

```
> argscape_load load --file <filename>
```

or, with sample and/or node locations, using

```
> argscape_load load-with-locations --file <filename> --sample-csv <filename> --node-csv <filename>
```

To view the complete set of file management commands, run

```
> argscape_load --help
```

S1.4.3 argscape_infer

The `argscape_infer` command unifies features for running spatial and temporal inference methods on loaded tree sequences. A simple command-line interface is provided for selecting files, inference methods, and output locations, which can be accessed by running

```
> argscape_infer
```

To view the complete set of spatiotemporal inference commands, run

```
> argscape_infer --help
```

S2 FastGaia Algorithm

`FastGaia` is an unpublished set of algorithms for inferring the geographic locations of ancestral nodes in a tree sequence given georeferenced samples. Drawing inspiration from `Gaia` and Wohns' midpoint approach [Wohns et al., 2022, Grundler et al., 2025], this approach aims to utilize more of the information encoded in the ARG than a simple midpoint approach, while running faster than `Gaia` by implementing a parallelizable greedy algorithm approach. The ease of incorporating `FastGaia` within the `ARGscape` framework demonstrates the flexibility of integrating new methods into `ARGscape`'s modular framework.

Like `Gaia`, `FastGaia` can operate in continuous space (no barriers to dispersal, Euclidean cost function) or discrete space (using a uniform transition cost between states or an input transition cost matrix). Note that `ARGscape` uses only the continuous-space version of the algorithm. The complete details of both `FastGaia` algorithms are provided below, along with the necessary notation.

`FastGaia` can be installed in Python environments running Python version 3.8 or later by running the command

```
> python -m pip install fastgaia
```

S2.1 `FastGaia` Algorithm Notation and Definitions

- T: Tree sequence with nodes V and edges E
- $n = |V|$: Number of nodes
- $t(u)$: Time (age) of node u

- $S \subset V$: Set of sample nodes with known locations/states
- $E(u)$: Set of edges where u is the parent
- $C(u) = \{v : (u, v) \in E\}$: Set of children of node u
- $P(u) = \{w : (w, u) \in E\}$: Set of parents of node u
- For edge $e = (u, v)$:
  - $s(e)$: Genomic span (right - left coordinates)
  - $b(e) = t(u) - t(v)$: Branch length (temporal distance)
- $\mathcal{E}_u$: Set of valid edges from parent u to children with known locations/states (used locally)
- ω_e: Weight assigned to edge e (continuous inference)

S2.2 `FastGaia` Algorithm S1: Continuous Location Inference

Input:

- Tree sequence $T = (V, E)$
- Sample locations $\mathcal{L}_S = \{\ell_u \in \mathbb{R}^d : u \in S\}$
- Boolean flags: w_{span}, w_{branch}

Output:

- Inferred locations $\mathcal{L} = \{\ell_u \in \mathbb{R}^d : u \in V\}$

Algorithm S1: Continuous Location Inference

Initialize $\ell_u \leftarrow \text{NaN} \in \mathbb{R}^d$ for all $u \in V$
$\ell_u \leftarrow \mathcal{L}_S(u)$ for all $u \in S$ ▷ Assign known sample locations
Partition nodes: $V_\tau = \{u \in V : t(u) = \tau\}$ for each unique time τ
Sort times: $\mathcal{T} = \{\tau_1, \tau_2, \ldots, \tau_k\}$ where $\tau_1 < \tau_2 < \cdots < \tau_k$
for each time $\tau \in \mathcal{T}$ **do** ▷ Process from present to past
  for each node $u \in V_\tau$ **do** ▷ Parallel processing possible
    if $u \in S$ **then**
      continue ▷ Sample location already known
    end if
    $\mathcal{E}_u \leftarrow \{e = (u, v) : v \in C(u) \text{ and } \ell_v \neq \text{NaN}\}$
    if $\mathcal{E}_u = \emptyset$ **then**
      $\ell_u \leftarrow \text{NaN}$ ▷ No valid children
      continue
    end if
    ▷ Compute weighted average location
    for each edge $e = (u, v) \in \mathcal{E}_u$ **do**
      $\omega_e \leftarrow 1.0$
      if $w_{\text{span}} = \text{True}$ **then**
        $\omega_e \leftarrow s(e)$
      end if
      if $w_{\text{branch}} = \text{True}$ **then**

$\omega_e \leftarrow \omega_e \cdot \frac{1}{b(e)}$ ▷ Inverse branch length
end if
end for
$W \leftarrow \sum_{e \in \mathcal{E}_u} \omega_e$
if $W = 0$ **then**
$\ell_u \leftarrow \frac{1}{|\mathcal{E}_u|} \sum_{e=(u,v) \in \mathcal{E}_u} \ell_v$ ▷ Equal weights fallback
else
$\ell_u \leftarrow \frac{1}{W} \sum_{e=(u,v) \in \mathcal{E}_u} \omega_e \cdot \ell_v$ ▷ Weighted average location of children
end if
end for
end for
return $\mathcal{L} = \{\ell_u : u \in V\}$

Weight Formula: For edge $e = (u, v)$ connecting parent u to child v:

$$\omega_e = \begin{cases} s(e) \cdot \frac{1}{b(e)} & \text{if } w_{\text{span}} \wedge w_{\text{branch}} \\ s(e) & \text{if } w_{\text{span}} \wedge \neg w_{\text{branch}} \\ \frac{1}{b(e)} & \text{if } \neg w_{\text{span}} \wedge w_{\text{branch}} \\ 1 & \text{if } \neg w_{\text{span}} \wedge \neg w_{\text{branch}} \end{cases}$$

Location Update:

$$\ell_u = \frac{\sum_{v \in C(u)} \omega_{(u,v)} \cdot \ell_v}{\sum_{v \in C(u)} \omega_{(u,v)}}$$

S2.3 `FastGaia` Algorithm S2: Discrete State Inference

Input:

- Tree sequence $T = (V, E)$
- Sample states $\mathcal{S}_S = \{\sigma_u \in \{1, 2, \ldots, m\} : u \in S\}$
- Cost matrix $M \in \mathbb{R}^{m \times m}$ (optional): M_{ij} = cost of transition from state i to state j

Output:

- Inferred states $\mathcal{S} = \{\sigma_u \subseteq \{1, \ldots, m\} : u \in V\}$ (set-valued to account for ties)

Algorithm S2: Discrete State Inference

Initialize $\sigma_u \leftarrow \emptyset$ for all $u \in V$
$\sigma_u \leftarrow \{\mathcal{S}_S(u)\}$ for all $u \in S$ ▷ Assign known sample states
Determine state space: $\Sigma = \{1, 2, \ldots, m\}$
Partition nodes: $V_\tau = \{u \in V : t(u) = \tau\}$ for each unique time τ
Sort times: $\mathcal{T} = \{\tau_1, \tau_2, \ldots, \tau_k\}$ where $\tau_1 < \tau_2 < \cdots < \tau_k$
for each time $\tau \in \mathcal{T}$ **do** ▷ Process from leaves to root
    for each node $u \in V_\tau$ **do** ▷ Parallel processing possible
        if $u \in S$ **then**
            continue ▷ Sample state already known
        end if
        ▷ Compute cost for each candidate state
        for each candidate state $\alpha \in \Sigma$ **do**

$\mathcal{C}_{\text{child}} \leftarrow 0$
for each edge $e = (u, v)$ where $v \in C(u)$ and $\sigma_v \neq \emptyset$ **do**
for each state $\beta \in \sigma_v$ **do**
$c \leftarrow M_{\alpha,\beta}$ if M provided, else $c \leftarrow 1$
$\mathcal{C}_{\text{child}} \leftarrow \mathcal{C}_{\text{child}} + c \cdot s(e) \cdot b(e)$
end for
end for
end for
$c^* \leftarrow \min_{\alpha \in \Sigma} \mathcal{C}_{\text{child}}$
$\sigma_u \leftarrow \{\alpha \in \Sigma : \mathcal{C}_{\text{child}} = c^*\}$ ▷ All optimal states
end for
end for
return $\mathcal{S} = \{\sigma_u : u \in V\}$

Cost Formula: For node u and candidate state α:

$$\mathcal{C}(u, \alpha) = \sum_{v \in C(u)} \sum_{\beta \in \sigma_v} c(\alpha, \beta) \cdot s_{uv} \cdot b_{uv}$$

where the transition cost function is

$$c(i, j) = \begin{cases} M_{ij}, & \text{if a cost matrix } M \text{ is provided} \\ 1, & \text{otherwise (uniform cost)} \end{cases}$$

State Assignment:

$$\sigma_u = \arg\min_{\alpha \in \Sigma} \mathcal{C}(u, \alpha)$$

Note: σ_u may include multiple states if there are ties for the minimum cost.

S3 2D Visualization Tip Ordering Algorithms

While all 2D `ARGscape` visualizations are fully interactive and customizable, we also provide a diverse set of tree sequence-specific tip ordering algorithms designed to clarify complex, tangled graph layouts. The available tip ordering algorithms are detailed below. Ideal choice of tip ordering algorithm will vary on a case-by-case basis, with no clear general rules for which to choose in what scenario. However, especially for very complex graphs, `dagre-d3` mode will often produce the clearest graphs.

S3.1 Numeric

The numeric tip ordering algorithm places sample nodes along the x-axis in order of increasing node ID. Ancestral nodes are placed using a force-directed simulation. This ordering will often result in complex and tangled visualizations.

S3.2 First Tree

The first-tree tip ordering algorithm places sample nodes along the x-axis in the order of a minlex postorder traversal of the first local tree in the tree sequence. This order is generated automatically by `tskit` [Kelleher et al., 2016, Ralph et al., 2020, Wong et al., 2024], and is

the same ordering algorithm used by `tskit_arg_visualizer` [Kitchens & Wong, 2025].

S3.3 Center Tree

The center-tree tip ordering algorithm places sample nodes along the x-axis in the order of a minlex postorder traversal of the middle local tree in the tree sequence. If only one local tree is available, this is the same order as in first-tree. If two trees meet the "center" criteria, the first is used.

S3.4 Consensus

The consensus tip ordering algorithm orders sample nodes along the x-axis according to a majority vote by minlex postorder traversals across K local trees in the tree sequence, where K scales with number of local trees, and is bounded by $[1, 50]$. The K local trees are selected from evenly spaced genomic positions across the tree sequence. If only one local tree is available, this is the same order as in first-tree or center-tree.

S3.5 Ancestral Path

The ancestral path tip ordering algorithm aims to group sample nodes by shared ancestry and similar time to coalescence. It creates groups of samples that coalesced at similar times, then places groups with more recent shared ancestry closer to the center of the graph. This aims to minimize path crossings by putting groups with deeper ancestry – and therefore longer edges – towards the outside of the graph.

S3.6 Coalescence

The coalescence tip ordering algorithm orders sample nodes along the x-axis in decreasing order of time to coalescence.

S3.7 Dagre-d3

When `dagre-d3` mode is enabled, all nodes in the graph are placed by the `dagre-d3` React library using an edge-crossing minimization algorithm. The force-directed simulation is disabled when this mode is active.

References

Grundler, MC, Terhorst, J, Bradburd, GS. A geographic history of human genetic ancestry. *Science* 2025; 387: 1391–1397.

Kelleher, J, Etheridge, AM, McVean, G. Efficient coalescent simulation and genealogical analysis for large sample sizes. *PLoS Comput Biol* 2016; 12: e1004842.

Kitchens, J, Wong, Y. tskit_arg_visualizer: interactive plotting of ancestral recombination graphs. *arXiv* 2025; doi: arXiv:2508.03958

Ralph, P, Thornton, K, Kelleher, J. Efficiently summarizing relationships in large samples: A general duality between statistics of genealogies and genomes. *Genetics* 2020; 215: 779–797.

Wohns, AW, Wong, Y, Jeffery, B, et al. A unified genealogy of modern and ancient genomes. *Science* 2022; 375: eabi8264.

Wong, Y, Ignatieva, A, Koskela, J, et al. A general and efficient representation of Ancestral Recombination Graphs. *Genetics* 2024; 228: iyae100.
